# Cardiac Structure and Function in Cushing's Syndrome: A Cardiac Magnetic Resonance Imaging Study

**DOI:** 10.1210/jc.2014-1783

**Published:** 2014-08-05

**Authors:** Peter Kamenický, Alban Redheuil, Charles Roux, Sylvie Salenave, Nadjia Kachenoura, Zainab Raissouni, Laurent Macron, Laurence Guignat, Christel Jublanc, Arshid Azarine, Sylvie Brailly, Jacques Young, Elie Mousseaux, Philippe Chanson

**Affiliations:** Faculté de Médecine (P.K., S.S., S.B., J.Y., P.C.), Univ Paris-Sud, F-94276 Le Kremlin Bicêtre, France; Faculté de Médecine (A.R., N.K.), Sorbonne Universités, Université Pierre et Marie Curie Univ Paris 06, F75006 Paris, France; Faculté de Médecine (E.M.), Université Paris Descartes, F75006 Paris, France; Service d'Endocrinologie et des Maladies de la Reproduction (P.K., S.S., J.Y., P.C.) and Service de Pharmacogénétique (S.B.), Biochimie Moléculaire et Hormonologie, Hôpital de Bicêtre, Assistance Publique-Hôpitaux de Paris, F-94275 Le Kremlin Bicêtre, France; Département d'Imagerie Cardiovasculaire (A.R.) and Service d'Endocrinologie (C.J.), Hôpital Pitié-Salpêtrière, Assistance Publique-Hôpitaux de Paris, F75013 Paris, France; Service de Radiologie Cardiovasculaire (C.R., L.M., A.A., Z.R., E.M.), Hôpital Européen George Pompidou, Assistance Publique-Hôpitaux de Paris, F75015 Paris, France; Service d'Endocrinologie (L.G.), Hôpital Cochin, Assistance Publique-Hôpitaux de Paris, F75006 Paris, France; U693 (P.K., J.Y., S.B., P.C.), Institut National de la Santé et de la Recherche Médicale (INSERM), F-94276 Le Kremlin Bicêtre, France; Unité Mixte de Recherche 7371 and Unité Mixte de Recherche en Santé 1146 (A.R., N.K.), Laboratoire d'Imagerie Biomédicale, ICAN Imaging Core Lab, INSERM, F-75013 Paris, France

## Abstract

**Background:**

Patients with Cushing's syndrome have left ventricular (LV) hypertrophy and dysfunction on echocardiography, but echo-based measurements may have limited accuracy in obese patients. No data are available on right ventricular (RV) and left atrial (LA) size and function in these patients.

**Objectives:**

The objective of the study was to evaluate LV, RV, and LA structure and function in patients with Cushing's syndrome by means of cardiac magnetic resonance, currently the reference modality in assessment of cardiac geometry and function.

**Methods:**

Eighteen patients with active Cushing's syndrome and 18 volunteers matched for age, sex, and body mass index were studied by cardiac magnetic resonance. The imaging was repeated in the patients 6 months (range 2–12 mo) after the treatment of hypercortisolism.

**Results:**

Compared with controls, patients with Cushing's syndrome had lower LV, RV, and LA ejection fractions (*P* < .001 for all) and increased end-diastolic LV segmental thickness (*P* < .001). Treatment of hypercortisolism was associated with an improvement in ventricular and atrial systolic performance, as reflected by a 15% increase in the LV ejection fraction (*P* = .029), a 45% increase in the LA ejection fraction (*P* < .001), and an 11% increase in the RV ejection fraction (*P* = NS). After treatment, the LV mass index and end-diastolic LV mass to volume ratio decreased by 17% (*P* < .001) and 10% (*P* = .002), respectively. None of the patients had late gadolinium myocardial enhancement.

**Conclusion:**

Cushing's syndrome is associated with subclinical biventricular and LA systolic dysfunctions that are reversible after treatment. Despite skeletal muscle atrophy, Cushing's syndrome patients have an increased LV mass, reversible upon correction of hypercortisolism.

Cardiovascular complications are a major cause of morbidity and mortality in patients with Cushing's syndrome (1–3). Patients with Cushing's syndrome are at increased risk of cardiovascular events, which does not fully normalize after remission (4–7). Increased blood pressure (BP), glucose intolerance or diabetes, central obesity, and metabolic syndrome (8) together with chronic hypokalemia (9) and a direct toxic effect of cortisol can all affect cardiac structure and function (2). Overt dilated cardiomyopathy with congestive heart failure is currently very rare, thanks to improved management of hypercortisolism and the use of antihypertensive drugs (eg, angiotensin converting enzyme inhibitors) with cardioprotective effects (10–12). However, subclinical structural and functional cardiac alterations are nearly always present but underdiagnosed.

To date, descriptions of cardiac structure and function in patients with Cushing's syndrome have been limited to two-dimensional (2D) echocardiography and restricted to the left ventricle (LV). A few studies have shown LV hypertrophy, concentric remodeling, and reduced systolic and diastolic performance (13–19). LV dysfunction was found to be associated with myocardial fibrosis in a single ultrasound (US) study (18) and was potentially reversed by normalization of the cortisol excess (17). One important limitation of these US studies is that the measurement of LV volumes and mass by 2D echocardiography requires assumptions to be made concerning LV geometry, introducing a source of inaccuracy and variability (20). In addition, patients with central obesity are especially prone to suboptimal image quality and off-axis images, which may limit the precision of echo-based calculations of ventricular mass and volumes.

In contrast, cardiac magnetic resonance imaging (CMR) provides a highly accurate and comprehensive assessment of cardiac geometry and function. CMR provides regional myocardial wall thickness, myocardial mass, and atrial and ventricular volumes based on precise delineation of myocardial borders without the need for geometric assumptions (21, 22). In addition, late gadolinium-enhanced CMR can depict dense myocardial fibrosis (23). The aim of this study was to characterize the consequences of cortisol excess on cardiac structure and function by means of CMR, comparing patients with Cushing's syndrome with healthy controls matched for age, gender, and body mass index (BMI), and evaluating the reversibility of cardiac abnormalities after the effective treatment of hypercortisolism.

## Materials and Methods

### Patients

Patients were recruited in the Endocrinology Department of a tertiary referral center from September 2009 to March 2013. Patients aged 15–60 years were eligible if they had active endogenous Cushing's syndrome (newly diagnosed or uncontrolled after first surgery). Cushing's syndrome was diagnosed according to the usual clinical and biological criteria, including elevated urinary free cortisol (UFC) excretion, loss of the circadian plasma cortisol pattern, and lack of cortisol suppression during the overnight 1-mg dexamethasone suppression test (1).

### Healthy volunteers

Healthy asymptomatic volunteers free of overt cardiovascular disease and risk factors for atherosclerosis, such as smoking, diabetes, dysplipidemia, and hypertension, were recruited by advertisement and matched with the patients for age, sex, and BMI.

All the patients and volunteers gave their written informed consent, and the study protocol was approved by the Paris-Sud Ethics Committee (Le Kremlin-Bicêtre, France). All procedures conformed to the Declaration of Helsinki.

### Investigations

Before cardiac investigations, the patients were admitted to the Endocrinology Department of our institution for clinical and biochemical investigations. BP (mean value of three measurements made at 2 min intervals after 30 min of rest in the sitting position) was determined with an automatic validated BP recorder (Press Mate BP 8800; Colin Co). The reported clinical BP is the mean value obtained in the 3 days preceding CMR. Plasma fasting glucose, triglycerides, total, low-density lipoprotein (LDL), and high-density lipoprotein (HDL) cholesterol, potassium and glycated hemoglobin (HBA_1_) were measured with standard procedures. In patients not known to have diabetes mellitus, glucose metabolism was assessed by a standard oral glucose tolerance test (OGTT). In the OGTT, the plasma glucose concentration was measured 2 hours after an oral intake of 75 g of glucose. Diabetes mellitus and impaired glucose tolerance were diagnosed according to the usual diagnostic criteria (24).

The repeat CMR evaluation (second evaluation) was performed when successful treatment was achieved, with clinical and biological remission of hypercortisolism. Patients were considered in remission after treatment if they presented either with secondary adrenal insufficiency, with low morning serum cortisol concentrations and low UFC excretion rates or with eucortisolism, with normal UFC excretion rates (<65 μg per 24 hours or < 179 nmol per 24 h), normal overnight suppression of serum cortisol after the administration of 1 mg dexamethasone and normal sleeping midnight serum cortisol concentration (<2 μg/dL or 55 nmoL/L for both) (25). The period elapsed in the remission of hypercortisolism needed to be at least 2 months and no more than 12 months.

### Assays

Plasma glucose, potassium, total cholesterol, LDL cholesterol, HDL cholesterol, triglycerides, and HBA_1_c were determined with standard analytical methods. UFC excretion was measured with a specific RIA using polyclonal antibodies as previously reported (26). Baseline UFC excretion is reported as the average of individual determinations in three consecutive daily urine samples. The normal range of UFC excretion is 10–65 μg per 24 hours (27–179 nmol per 24 hours). Serum cortisol concentrations were measured with a chemiluminescent competitive immunoassay using a polyclonal antibody as previously reported (26).

### Cardiac magnetic resonance imaging (MRI)

All CMR examinations were performed in a tertiary academic referral hospital on a 1.5T (GEMS) MRI scanner using a dedicated torso 8-element phased array coil and electrocardiographic gating. All acquisitions were done with breath holding. Cine images were acquired using a steady-state free precession sequence and covered the heart in axial views (10–12 slice levels) and in cardiac short-axis views covering the entire right and left ventricles (10–12 slice levels) with the following parameters: echo time, 1.5 msec; repetition time, 3.5 msec; slice thickness, 8 mm; spacing, 10 mm; acquisition matrix, 224 × 192; spatial resolution, 0.78 mm; and number of phases, 40. Late gadolinium enhancement of the myocardium was studied with a T1-weighted 2D inversion recovery sequence on long- and short-axis end-diastolic views of the LV, 10–15 minutes after iv infusion of 0.2 mmol/kg of a gadolinated contrast agent (Gd-DTPA, Dotarem; Guerbet) with the following parameters: echo time, 1.5 msec; repetition time, 5.3 msec; slice thickness, 8 mm; spacing, 10 mm; acquisition matrix, 224 × 160; spatial resolution, 0.86 mm; and inversion time, 250 msec. These investigations were repeated in the same conditions during the patients' usual follow-up visits after effective treatment of Cushing's syndrome.

### Image analysis

End-diastolic and end-systolic LV and right ventricular (RV) myocardial contours were generated on short-axis images by using semiautomated software (Qmass; MEDIS) by an experienced reader and were all validated by consensus after adjustment if necessary by an expert reader for all subjects. Endocardial contours were used to estimate ventricular end-diastolic and end-systolic volumes by the disk summation method. Similarly, end-diastolic LV myocardial contours were used to measure LV myocardial volume and, subsequently, LV mass (grams) as LV myocardial volume × myocardial density (1.06 g/mL). Left atrial end-diastolic and end-systolic endocardial contours were traced manually on axial cine images with the same method and used to measure left atrial (LA) volumes. LV and RV stroke volumes (milliliters) were then calculated as end-diastolic volume minus end-systolic volume, and ejection fractions were calculated (in percentages) as stroke volume/end-diastolic volume. Segmental wall thickness (millimeters) was measured as the mean segmental thickness on each American Heart Association segment (27) using epicardial and endocardial LV contours and the centerline method with 100 chords per slice level. Average wall thickness was calculated for the basal, mid-LV, and apical slices. LV concentricity was assessed by calculating the LV end-diastolic mass to volume ratio. Cardiac volumes and LV mass were indexed to BSA.

### Statistical analyses

The LV and RV ejection fractions were used as the main measures of ventricular systolic performance. Continuous variables are expressed as the median and interquartile range. Differences between the controls and patients with active Cushing's syndrome were analyzed with the Mann-Whitney test. Treatment-induced changes in the patients are expressed as baseline-adjusted changes from baseline (pretreatment value minus posttreatment value/pretreatment value × 100%). The effects of Cushing's syndrome treatment were assessed with a Wilcoxon's rank-signed test. Univariate associations between clinical variables (age, gender, height, weight, BMI, systolic and diastolic BP) and biological variables (fasting glucose, HBA_1_c, UFC, morning plasma cortisol) and the LV, RV, and LA ejection fractions, the LV mass index and mean wall thickness were analyzed with the Spearman rank correlation, as were associations with treatment-induced changes in these parameters. To evaluate the influence of selected predictive variables on treatment-induced changes in the LV, RV, and LA ejection fractions, the LV mass index and mean wall thickness, we used multiple linear regression models with adjustment for age, gender, BMI, and systolic BP, testing each independent variable individually. Significance was assumed at *P* < .05. Stata 11SE (StataCorp) and SAS 9.1 software (SAS Institute) were used for statistical analyses.

## Results

### Baseline clinical and biological characteristics

Eighteen patients with active Cushing's syndrome (16 women and two men) with a median age of 35 years (range 15–59 y) were included. Fourteen patients had newly diagnosed Cushing's syndrome. Four patients had previously been treated by pituitary surgery and had developed recurrent hypercortisolism before entering the study. Fifteen patients had Cushing's disease, one had ectopic ACTH secretion, and two had cortisol-secreting adrenal adenomas. The patients' baseline clinical and hormonal characteristics are summarized in Supplemental Table 1. One patient (number 16) had New York Heart Association (NYHA) II-III dyspnea. The other patients had no symptoms of heart failure (NYHA I). Six patients were obese (BMI > 30 kg/m^2^), six were overweight (25 < BMI < 30 kg/m^2^), and six had a normal BMI (<25 kg/m^2^). Nine patients had arterial hypertension (World Health Organization/International Society of Hypertension definition), seven of whom were on antihypertensive treatment. Five patients were diabetic (World Health Organization/International Diabetes Foundation definition), of whom three required oral antidiabetic drugs. The OGTT revealed impaired glucose tolerance in another four patients. Five patients had hypertriglyceridemia and one had hypercholesterolemia. Three patients were on statin therapy and three patients required potassium supplementation.

As expected, sex, age, and BMI were similar in the patients and controls. Systolic and diastolic BPs were higher in the patients than in the controls (Table 1).

**Table 1.** Baseline Clinical Parameters of the Control Subjects and Cushing's Syndrome Patients

	Controls (n = 18)	Patients (n = 18)	*P* Value
Age, y	40.5 [24.2; 51.5]	35.0 [26.2; 47.2]	NS
Sex, female, %	13 (72%)	16 (89%)	NS
Body surface area, m^2^	1.73 [1.66; 1.96]	1.80 [1.68; 2.01]	NS
BMI, kg/m^2^	24.5 [21.8; 28.1]	27.5 [22.2; 33.0]	NS
Systolic BP, mm Hg	112 [108; 117]	120 [113; 130]	.009
Diastolic BP, mm Hg	67 [64; 72]	77 [72; 88]	<.001
Heart rate, beats/min	68 [64; 73]	72 [65; 83]	NS

Abbreviation: NS, not significant. Data are expressed as the median and interquartile range.

### Treatment of Cushing's syndrome

The second evaluation was performed a median of 6 months (range 2–12 mo) after correction of the glucocorticoid excess. Ten patients underwent transphenoidal surgery, four received steroidogenesis inhibitors, and one received both transphenoidal surgery and medical treatment. The patient with ectopic ACTH secretion was prepared with medical therapy before curative thoracic surgery. Both patients with cortisol-secreting adrenal adenomas were cured by unilateral adrenalectomy. Clinical and biological remission of hypercortisolism was obtained in all the patients (Table 2). Fourteen operated patients had secondary corticotroph deficiency after successful treatment of hypercortisolism and received effective hydrocortisone replacement therapy (20 mg daily).

**Table 2.** Clinical, Biochemical, and Hormonal Parameters of Cushing's Syndrome Patients Before and After Successful Treatment

	Patients		*P* Value
	Before T (n = 18)	After T (n = 17)	
Clinical parameters			
Body surface area, m^2^	1.80 [1.68; 2.01]	1.77 [1.69; 1.98]	NS
BMI, kg/m^2^	27.5 [22.2; 33.0]	25.5 [21.0; 30.3]	.020
Systolic BP, mm Hg	120 [113; 130]	114 [100; 123]	.029
Diastolic BP, mm Hg	77 [72; 88]	72 [63; 79]	.020
Heart rate, beats/min	72 [65; 83]	64 [61; 71]	NS
Biochemical parameters (plasma)			
Fasting glucose, mmol/L	4.80 [4.45; 5.50]	4.30 [4.05; 4.50]	.002
Glucose 2 h after OGTT, mmol/L	8.75 [6.85; 10.5]	5.50 [4.60; 7.40]	.027
HBA_1c_, %	6.00 [5.80; 6.40]	5.45 [5.20; 5.82]	.004
Triglycerides, mmol/L	1.06 [0.67; 1.83]	1.00 [0.83; 1.99]	NS
Total cholesterol, mmol/L	5.39 [4.68; 5.75]	5.34 [4.66; 6.02]	NS
LDL cholesterol, mmol/L	3.02 [2.48; 3.43]	3.24 [2.54; 3.95]	NS
HDL cholesterol, mmol/L	1.67 [1.53; 1.95]	1.36 [1.23; 1.61]	.029
K, mmol/L	4.00 [3.67; 4.30]	4.00 [3.82; 4.40]	NS
Hormonal parameters			
UFC, μg per 24 h	373 [178; 807]	6.0 [5.0; 19.0]	<.001
Morning plasma cortisol, μg/dL	24.6 [19.4; 33.2]	4.8 [1.3; 8.3]	.002
Midnight plasma cortisol, μg/dL	18.1 [14.0; 22.5]	1.1 [1.0; 1.9]	.002

Abbreviations: K, potassium; T, treatment. Data are expressed as the median and interquartile range.

Data for 17 patients were included in the analyses of the response to treatment. Patient 18 was excluded from further investigations because of pregnancy. In addition, data for the subgroup of 13 patients with surgical remission and secondary corticotroph deficiency also were analyzed separately. The impact of correction of the glucocorticoid excess on clinical and biological parameters is summarized in Table 2. BMI and systolic and diastolic BPs fell significantly after treatment, whereas the heart rate was unchanged. Antihypertensive therapy could be discontinued in four patients and reduced in another two. Fasting glucose levels, glucose levels 2 hours after the OGTT, and HBA_1_c levels also fell after treatment. Diabetes mellitus and impaired glucose tolerance reversed in all treated patients. Triglyceride, total cholesterol, and LDL cholesterol levels did not change, whereas HDL cholesterol levels fell after treatment. Statins could be discontinued in one patient.

### Baseline cardiac parameters in patients and controls

**Figure 1.**
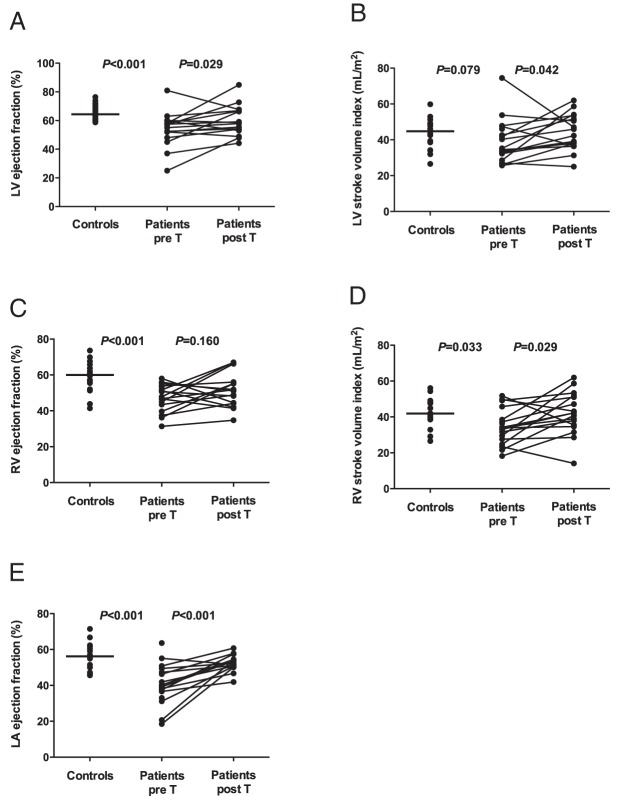
Comparison of LV ejection fractions (A), LV stroke volume indexes (B), RV ejection fractions (C), RV stroke volume indexes (D), and LA ejection fractions between control subjects and Cushing's syndrome patients before and after treatment. Pre T, before treatment; post T, after treatment.

**Figure 2.**
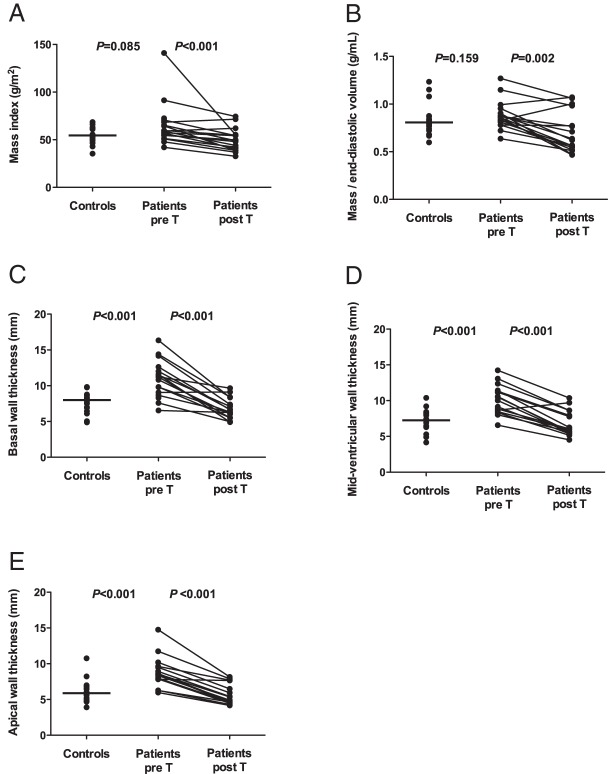
Comparison of LV mass indexes (A), LV mass to end-diastolic volume ratios (B), basal wall thickness (C), midventricular wall thickness (D), and apical wall thickness (E) in control subjects and Cushing's syndrome patients before and after treatment. Pre T, before treatment; post T, after treatment.

LV, RV, and LA parameters in the patients and controls are compared in Table 3. Patients and controls had similar LV end-diastolic volumes, whereas LV end-systolic volumes were higher in the patients. Consequently, the patients had lower LV ejection fractions (*P* < .001) and tended to have a smaller LV stroke volume index (*P* = .08) (Figure 1). In addition, compared with controls, patients had markedly increased end-diastolic LV segmental thickness in the basal, mid-LV and apical slices (all *P* < .001), and also a trend toward higher LV mass (Figure 2). Only one patient (number 16) had LV hypertrophy according to conventional CMR thresholds defining LV hypertrophy (28). At baseline, controls and patients had similar LV mass to end-diastolic volume ratio (Figure 2). Differences in RV parameters between controls and patients at baseline were similar to the differences in LV parameters. RV end-diastolic volumes were similar in the patients and controls, but the patients had higher RV end-systolic volumes (*P* = .012), lower RV stroke volumes (*P* = .033), and lower RV ejection fractions (*P* < .001) (Figure 1). Maximal LA volumes were similar in the patients and controls, but minimal LA volumes after atrial contraction were significantly larger in patients than controls at baseline (*P* = .042). Consequently, LA ejection fractions (Figure 1) were markedly lower in patients than controls (*P* < .001). In a univariate analysis, the patients' baseline CMR LV and RV function estimates did not correlate with their clinical or biological variables. The LA ejection fraction was associated only with elevated fasting glucose (r = 0.72, *P* = .043), and the LV mass index was associated only with systolic BP (r = 0.72, *P* = .045). No delayed myocardial enhancement was observed.

**Table 3.** LA, LV, and RV Parameters Assessed by CMR in Control Subjects and in Cushing's Syndrome Patients Before and After Treatment

	Controls (n = 18)	Patients Pre T (n = 18)	Patients Post T (n = 17)	*P* Value Controls vs Pre T Patients	*P* Value Effect of T
Left ventricular parameters					
End-diastolic volume index, mL/m^2^	67.9 [52.5; 77.6]	68.8 [62.5; 88.8]	71.7 [61.1; 87.9]	NS	NS
End-systolic volume index, mL/m^2^	22.4 [16.7; 29.4]	31.8 [24.2; 37.4]	31.3 [21.4; 36.6]	.002	NS
Stroke volume index, mL/m^2^	44.8 [37.5; 49.7]	34.9 [31.4; 46.4]	42.3 [37.3; 52.6]	.079	.042
Ejection fraction, %	64.3 [60.9; 69.6]	55.0 [51.0; 58.4]	57.8 [53.3; 66.7]	<.001	.029
Mass index, g/m^2^	53.6 [50.1; 62.1]	59.1 [53.8; 69.0]	49.1 [39.8; 58.5]	.085	<.001
Mass to end-diastolic volume, g/mL	0.81 [0.74; 0.87]	0.86 [0.80; 0.92]	0.62 [0.53; 0.88]	NS	.002
Basal wall thickness, mm	7.99 [6.78; 8.61]	11.2 [8.96; 12.2]	6.30 [5.52; 7.87]	<.001	<.001
Midventricular wall thickness, mm	7.26 [6.33; 8.21]	10.3 [8.43; 11.3]	5.97 [5.54; 8.28]	<.001	<.001
Apical wall thickness, mm	5.90 [5.16; 6.68]	8.45 [7.86; 9.52]	4.90 [4.61; 7.07]	<.001	<.001
Right ventricular parameters					
End-diastolic volume index, mL/m^2^	69.6 [60.1; 80.0]	73.7 [62.8; 84.7]	83.5 [61.5; 91.1]	NS	NS
End-systolic volume index, mL/m^2^	27.1 [21.6; 35.3]	36.6 [32.6; 42.5]	39.2 [28.6; 46.1]	.012	NS
Stroke volume index, mL/m^2^	41.9 [37.1; 48.3]	34.1 [26.9; 40.3]	42.2 [35.1; 51.5]	.033	.029
Ejection fraction, %	60.0 [54.6; 64.3]	49.3 [42.5; 53.4]	51.1 [44.3; 55.6]	<.001	NS
Left atrial parameters					
Maximal volume index, mL/m^2^	37.7 [29.4; 48.5]	33.2 [27.6; 39.2]	33.8 [31.0; 42.4]	NS	NS
Minimal volume index, mL/m^2^	15.6 [13.1; 20.7]	20.8 [15.5; 24.7]	15.8 [13.9; 21.2]	.042	.004
Ejection fraction, %	56.1 [48.6; 61.2]	39.2 [34.8; 48.4]	52.0 [50.5; 55.2]	<.001	<.001

Abbreviations: Pre T, before treatment; post T, after treatment; T, treatment. Data are expressed as the median and interquartile range.

### Changes in cardiac parameters after treatment of Cushing's syndrome

**Figure 3.**
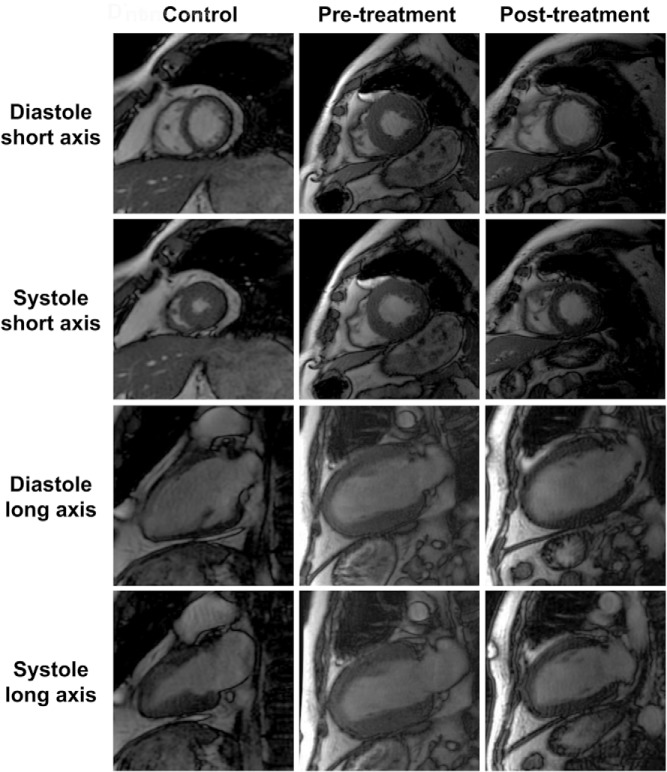
Steady-state free precession cine end-diastolic and end-systolic short- and long-axis images before and after treatment of Cushing's syndrome in patient 16 compared with a matched control subject. Note the global LV wall thickening without end-diastolic LV or RV dilatation and the decreased biventricular ejection in this symptomatic patient (NYHA II-III) prior to treatment and the clear reversal of this phenotype after treatment.

Changes in LV, RV, and LA parameters after the treatment of Cushing's syndrome are presented in Table 3. Treatment did not significantly affect LV end-diastolic or end-systolic volumes. LV systolic function improved after treatment, as reflected by, respectively, 19% and 15% increases in LV stroke volume index (*P* = .042) and LV EF (*P* = .029) (Figure 1). Treatment of cortisol excess was also associated with a 17% reduction in the LV mass index (*P* < .001). Segmental myocardial wall thickness decreased by 37%, 34%, and 35% at the basal, mid-LV, and apical levels (all *P* < .001), respectively. LV mass/end-diastolic volume decreased by 10% (*P* < .001), pointing to a more eccentric LV geometry, because the decrease in LV mass was associated with a trend toward an increase in end-diastolic volume (Figure 2). Treatment-induced changes in RV parameters were similar to changes in LV parameters. RV end-diastolic volumes and end-systolic volumes were not affected by treatment, but RV stroke volumes and the RV EF increased by 29% (*P* = .033) and 11% (*P* = .16), respectively, reflecting the improvement in RV systolic function (Figure 1). Maximal LA volumes were unchanged by treatment, but minimal LA volumes fell by 16% (*P* = .004) and, consequently, the LA ejection fraction increased by 45% (*P* < .001) (Figure 1). The results for the subgroup of 13 patients with surgical remission and secondary corticotroph deficiency were similar to those found for all 17 patients, except for the increase in RV stroke volumes that did not reach statistical significance (*P* = .13) when the four nonsurgically treated patients were excluded from the analysis. The effects of Cushing's syndrome treatment on ventricular function and structure, as assessed by CMR in patient 16, are illustrated in Figure 3.

In a univariate analysis, treatment-induced changes in the LV, RV, and LA ejection fraction did not correlate with changes in any of the clinical or biological parameters, except for an association between increased RV ejection fractions and decreased systolic BP (r = 0.68, *P* = .042). The treatment-induced decrease in the LV mass index correlated only with the decrease in blood glucose (r = −0.82, *P* = .007). In a multivariate analysis with adjustment for age, gender, BMI, and systolic BP, the only individual variable significantly associated with the increase in the LV ejection fraction was the decrease in mean wall thickness (r^2^ = 0.77, *P* = .030). Independent correlates of the decrease in the LV mass index included decreased fasting glucose (r^2^ = 0.82, *P* < .001), decreased BMI (r^2^ = 0.61, *P* = .017), and decreased mean wall thickness (r^2^ = 0.60, *P* = .048). None of the patients had late gadolinium enhancement of the myocardium on follow-up CMR.

## Discussion

This is the first CMR study of cardiac structure and function in Cushing's syndrome. We made three important findings. First, compared with controls, patients with Cushing's syndrome had a subclinical decrease in biventricular systolic performance, associated with increased LV mass and LA dysfunction. Second, effective treatment of hypercortisolism improved the systolic performance of both ventricles and LA, reduced LV mass, and LV wall thickness and led to the regression of the concentric LV remodeling pattern. The treatment-related decrease in LV mass was independently associated with changes in glucose metabolism and BMI. Finally, late gadolinium enhancement of the myocardium was absent in all patients, suggesting the absence of dense replacement myocardial fibrosis in uncomplicated Cushing's syndrome.

This CMR study confirms and extends echocardiographic findings on LV systolic and diastolic dysfunction in patients with in Cushing's syndrome. Indeed, several echocardiographic studies have evaluated cardiac structure and function in patients with Cushing's syndrome, either in the active phase (13–16) or both before and after treatment in adult (17–19) and pediatric patients (29). However, echo-based evaluation of ventricular mass and volumes requires geometric assumptions that may limit its accuracy, especially in case of central obesity, which is frequent in Cushing's syndrome patients. Furthermore, US measurement of RV and LA volumes is challenging, and these studies almost exclusively focused on the LV (13–19). Here the cardiac consequences of cortisol excess were analyzed by CMR, allowing highly accurate, noninvasive assessment of cardiac geometry and function. In addition to LV parameters, we were able to analyze RV and LA structure and function. Patients with active Cushing's syndrome were compared with age-, sex-, and BMI-matched control subjects. The reversibility of cardiac abnormalities was evaluated after the effective treatment of hypercortisolism.

One of our main findings is that LV and RV systolic performance is diminished in active Cushing's syndrome and that it improves after successful treatment of hypercortisolism. Compared with controls, patients had lower LV and RV ejection fractions, which increased by 15% and 11%, respectively, after successful treatment. End-diastolic volumes were similar in the patients (both before and after treatment) and controls, ruling out marked preload differences (30). Previous echo-based studies showed no changes in LV volumes or ejection fraction but revealed subclinical alteration of LV systolic performance based on calculated parameters requiring geometric assumptions, such as decreased LV endocardial and midwall fractional shortening (14, 19). Recently decreased LV circumferential and longitudinal strain was found by 2D speckle tracking imaging (17, 18). It is noteworthy that, except for patient 16, the patients in this study were all asymptomatic (NYHA I). Subclinical biventricular systolic dysfunction may therefore be largely underestimated in patients with Cushing's syndrome. Careful evaluation of cardiac structure and function by means of high-precision quantitative imaging in a specialized setting is thus important for appropriate cardiovascular management of Cushing's syndrome patients.

In addition to biventricular systolic dysfunction, patients with Cushing's syndrome had a markedly lower LA ejection fraction than controls, pointing to diastolic LV dysfunction. This parameter showed a marked improvement after treatment. Diastolic LV dysfunction has previously been reported in terms of abnormal transmitral flow velocity patterns on Doppler US (14–16, 19) and as decreased diastolic myocardial strain rates on 2D speckle tracking (17, 18). This finding is important, given the adverse prognostic value of dilated LA, which could potentially promote atrial fibrillation, stroke, and heart failure in iatrogenic hypercortisolism (3).

This study further demonstrates that cortisol excess is associated with increased regional LV wall thickness. However, no significant increase in total LV mass was found, probably owing to the small number of patients we were able to study, this being a rare disease. The LV mass to end-diastolic volume ratio remained close to young healthy control values and lower than values associated with an adverse prognosis in the asymptomatic general population (30). However, treatment of Cushing's syndrome led to a 17% reduction in the LV mass index, associated with a noteworthy decrease in LV segmental myocardial wall thickness and a decrease in the LV mass to end diastolic volume ratio (28). Previous echo-based studies of Cushing's syndrome patients also showed an increased relative myocardial wall thickness of the LV (13) and an increased LV mass index (14, 17–19). LV hypertrophy was present in 24%–42% of patients in some of these studies, but echocardiographic assessment may overestimate these abnormalities. In our study, only one patient (number 16) had LV hypertrophy according to the conventional CMR threshold (28). Accurate evaluation of LV mass and remodeling are highly relevant for risk stratification and therapeutic management of patients with Cushing's syndrome because increased LV mass and LV concentric remodeling are independent risk factors for cardiovascular events, incident heart failure, and sudden death (30, 31).

The pathophysiology of cardiac hypertrophy and decreased systolic function in Cushing's syndrome remains unclear. The myocardium seems to be exempt from the generalized muscular atrophy seen in Cushing's syndrome, resulting from increased protein catabolism (1). Arterial hypertension, present in up to 75% of patients with Cushing's syndrome (1, 5), may partly explain the increased LV mass, but hypertrophy has also been described in patients with normal BP (13, 17, 19). In our study the LV mass index was related to baseline systolic BP, but no statistically significant correlation was found between the treatment-induced changes in LV mass index and BP. Other Cushing's syndrome-related cardiovascular risk factors such as visceral obesity, glucose intolerance, and dyslipidemia (1, 5) may also contribute to the elevated LV mass index. Indeed, we found that treatment-induced changes in plasma fasting glucose and BMI were independent correlates of the change in the LV mass index. However, patients with Cushing's syndrome have impaired systolic performance that is not observed in obese subjects, who have increased stroke volumes, an unchanged LV ejection fraction, and significantly higher LV mass values than Cushing patients (32). Lastly, the cortisol excess per se may exert a toxic effect on the heart, mediated directly through glucocorticoid and/or mineralcocorticoid receptors (33, 34), and indirectly by inducing the expression of adrenergic receptors (35).

Decreased systolic performance in Cushing's syndrome points to ultrastructural abnormalities in heart muscle, altering its contractility. Recently increased myocardial fibrosis, indirectly assessed by calibrated integrated backscatter, has been described in patients with Cushing's syndrome. Fibrosis was associated with systolic and diastolic dysfunction and regressed after treatment of hypercortisolism (18). In the present study, however, late gadolinium-enhanced CMR, the reference method for the noninvasive assessment of dense myocardial fibrosis (23), revealed no focal intramyocardial fibrosis or myocardial infarction scar either at baseline or during the follow-up in any of the patients. However, this does not preclude the presence of diffuse interstitial fibrosis. Cardiac steatosis, documented by magnetic resonance spectroscopy in patients with impaired glucose tolerance or diabetes mellitus (36), could also contribute to impairing the myocardial contractility in Cushing's syndrome patients, who frequently have visceral obesity and diabetes mellitus.

The heterogeneity of the treatments used to control Cushing's syndrome is a potential limitation of this study. However, the results of reevaluation analysis did not chance substantially after exclusion of patients controlled only by medical therapy. Furthermore, we cannot rule out a potential impact of hydrocortisone replacement on the cardiac structure and function in patients with surgical remission and secondary corticotroph insufficiency.

In conclusion, this CMR study shows that patients with Cushing's syndrome frequently have significant subclinical biventricular and LA systolic dysfunction and increased LV mass, in the absence of dense myocardial fibrosis. Successful treatment of the cortisol excess improves systolic ventricular and atrial performance and leads to regression of the LV mass and myocardial thickness, in parallel with an improvement in glucose metabolism and BMI. Structural and functional abnormalities of the myocardium occur early in Cushing's syndrome and should be actively investigated to guide strategies designed to prevent overt heart failure. Although endogenous Cushing's syndrome is a rare disease, our findings may also have implications for patients receiving long-term treatment with exogenous corticosteroids, a common clinical situation.
